# Comments on “Low-Cost Alternative External Rotation Shoulder Brace and Review of Treatment in Acute Shoulder Dislocations”

**DOI:** 10.5811/westjem.2015.3.25815

**Published:** 2015-04-29

**Authors:** Robert W. Jordan, Adnan Saithna

**Affiliations:** University Hospitals Coventry and Warwickshire, Coventry, United Kingdom; Southport and Ormskirk Hospitals NHS Trust

Lacy K, Cooke C, Cooke P, et al. Low-cost alternative external rotation shoulder brace and review of treatment in acute shoulder dislocations. *West J Emerg Med*. 2015;16(1):114–120.

To the Editor:

We read the paper of Lacy et al. (2015) with interest.^1^ The authors present a narrative review of the use of external rotation bracing in acute shoulder dislocations. One of the weaknesses of a narrative review is that it is more likely to be subject to reporting bias. In their review the authors focus on published studies that demonstrated successful outcomes. The first two originate from Itoi et al. whose 2003 randomized controlled trial popularized the concept of external rotation bracing in this patient group.^2^,^3^ However, their good results have not been replicated in three subsequent randomized controlled trials.^4^–^6^ The third study cited concluded that external rotation bracing was advantageous.^7^ However, the major confounding factor in that study was that the internal rotation group had a younger mean age. As this is a well-established risk factor for re-dislocation the results of this study should be interpreted with caution.^8^,^9^ Furthermore, a recent systematic review and meta-analysis also concluded that ER bracing is not advantageous.^10^,^11^ We feel that the narrative review in this publication does not provide a balanced overview of the clinical studies available and we question the value of external rotation in the management of these patients.

Lacy et al. also describe the production of a low-cost external rotation brace that is more cost effective than those commercially available. The image of the sling provided demonstrates that this device produces only a small degree of external rotation. A recent systematic review demonstrated that reduction of the labrum is only achieved in 35% of cases when the arm is positioned in over 30 degrees of external rotation.^12^ However, the clinical studies previously discussed^3^–^5^,^7^ only achieved 10 to 20 degrees of rotation, and the illustration of the described technique in this paper suggests even less was achieved with this alternative brace. As a result, its effectiveness in achieving labral reduction and shoulder stability cannot be extrapolated from previous studies where a higher degree of rotation was obtained. An additional factor not addressed is the acceptability of the splint to patients. External rotation bracing is extremely inconvenient and poorly tolerated. Its prescription is associated with poor compliance, which may limit effectiveness.^3^–^5^

In closing we commend the authors on their innovative thinking and consider their design to be a cost-effective alternative to commercially available external rotation braces for posterior dislocations. However, the lack of any clear advantage to external rotation bracing for anterior shoulder dislocations in previous systematic reviews and meta-analysis should limit the subjection of patients to this poorly tolerated brace. The cheaper, more readily available internal rotation sling remains the standard treatment for these patients.
